# Estimation of weight in adults from height: a novel option for a quick bedside technique

**DOI:** 10.1186/s12245-018-0212-9

**Published:** 2018-11-27

**Authors:** Daniel D. Kokong, Ishaya C. Pam, Ayuba I. Zoakah, Solomon S. Danbauchi, Emmanuel S. Mador, Barnabas M. Mandong

**Affiliations:** 1 Department of ORL-Head and Neck Surgery (ORL-HNS), College of Medicine, University of Jos, Jos University Teaching Hospital, PMB 2076, Jos, Plateau State Nigeria; 2 Department of Obstetrics and Gynaecology, College of Medicine, University of Jos, Jos University Teaching Hospital, Plateau state, Nigeria; 3 Department of Public Health, College of Medicine, University of Jos, Jos University Teaching Hospital, Plateau state, Nigeria; 4 Department of Internal Medicine, College of Medicine, University of Jos, Jos University Teaching Hospital, Plateau state, Nigeria; 5 Department of Human Anatomy, College of Medicine, University of Jos, Plateau state, Nigeria; 6 Department of Pathology, College of Medicine, University of Jos, Jos University Teaching Hospital, Plateau State, Nigeria

**Keywords:** Estimation of weight, Adults, Height, Novel technique, Critical care, ATLS

## Abstract

**Purpose:**

In critical care situations, there are often neither the means nor the time to weigh each patient before administering strict weight-based drugs/procedures. A convenient, quick and accurate method is a priority in such circumstances for safety and effectiveness in emergent interventions as none exists in adults while those available are complex and yet to be validated. We aimed to study the correlation and accuracy of a quick bedside method of weight estimation in adults using height.

**Method:**

The technique is estimated body weight—eBW(kg) = (*N* − 1)100, where ‘*N*’ is the measured height in metres.

Adult undergraduates were enrolled 10/09/2015. Their heights and weights were measured while the formula was used to obtain the estimated weight. The SPSS version 21.0, Chicago, IL, USA was utilised for data analysis.

**Results:**

We analysed 122 participants aged 21–38 years with height = 1.55 m–1.95 m. The actual body weight range = 48.0 kg–91.0 kg, mean = 65.3 kg ± 9.7 kg and S.E. = 2.0 while eBW = 55 kg–95 kg, mean = 69.1 kg ± 8.4 kg and S.E. = 1.5. On BMI classes, a positive predictive value of 94.7% for the ‘normal’ category and 95.5% for ‘overweight’.

Correlation coefficient at 99% confidence interval yielded (*r*) = + 1, (*P* = 0.000) while the linear regression coefficient (*r*^2^) = + 1 at 95% confidence interval (*P* = 0.000).

The strength of agreement/precision was established by the Bland-Altman plot at 95% ± 2 s (*P* = 0.000) and kappa statistic with value = 0. 618.

**Conclusion:**

This unprecedented statistical characterisation of the two weight estimate measures to have a good agreement scientifically proposes the utility of our method with the formula eBW(kg) = 100(*N*−1) in critical care and ATLS protocol.

## Background

Health care is characterised by a reliance on human operators who work with increasingly complex technology and variable levels of uncertainty. Errors are inevitable and may have serious consequences for life. Industries with similar characteristics, notably aviation, have developed methods of documenting and investigating risk that allows systematic efforts to reduce the frequency and severity of adverse events. The Harvard Medical Practice study by Brennan et al. [1] remains the benchmark for the comprehensive study on medical errors with the attendant implication in the health sector. In 1998/9, medical litigation in the UK with an estimated cost of £2.4 billion in potential liability of which 2.3% were medication errors was reported [2]. In the USA, medical errors are the third leading cause of death only behind heart disease and cancer [3] with drug-related morbidity and mortality cost-of-illness estimated at $177.4 billion annually. Medication error accounted for a fatality of 1% and a life-threatening complication in 12% in a study [4, 5].

Dosage errors can occur when medications are ordered, especially in emergency states which has been found to occur twice as common in the intensive care unit (ICU)/emergency setting than non-ICU [6, 7]. Measuring weight may be impossible, especially for critically ill-patients, dire emergency conditions and before anaesthesia [8, 9]. In the operating room, with stressful situation, patients may frequently arrive unconscious or with emergency condition and accurate weight measurement can be difficult or impossible to obtain [10]. To bridge this gap, medical personnel often depended largely on crude estimation methods using 70 kg for males and 60 kg for females in critical care situations [11] with their potential lethal consequences.

An overestimation of the patients’ weights will increase the calculated dosage of strict-weight-based drugs/measures with narrow margin of safety and may result in potentially life-threatening side-effects while weight underestimation would result to suboptimal dosaging; thus, inaccurate estimation of total body weight in critical state is potentially dangerous [6, 12]. Therefore, accurate estimation or measurement of weight in the critically ill is important in optimum clinical care thus the need for an estimation method that is near accurate to the ‘ideal’ or ‘actual’ body weight that is quick with ease of recall to minimise medication error.

In children, easy to apply formulas for estimating weights in emergency situations exist [13, 14]. In adults, however, various scholars had observed a relationship between height and weight centuries ago that led to diverse formulas which are often complex; such as the Lorenz/Crandell’s formulas and the use of tibial length [11, 15] among several others of which none is yet to be validated for application in emergency medicine.

This study was therefore designed to evaluate the correlation, accuracy, and strength of agreement of a Novel Quick Bedside technique to rapidly estimate weights in adults using readily obtainable stable anthropometric measurement—the height. The new method could be of immense value in Critical Care Medicine and in the Advanced Trauma Life Support (ATLS) protocol.

## Material and methods

### Study population

All final year (fifth year) clinical medical students of the College of Medicine, University of Jos, Plateau State, Nigeria, aged ≥ 18 years that met the inclusion criteria who gave consent were enrolled for the study on 10/09/2015.

### Study design

Cross-sectional pilot cohort study.

### Setting

Academic public tertiary institution.

### Inclusion/ exclusion criteria

All final year (fifth year) medical students ≥ 18 years who consent. Gravid females were excluded.

### Sampling method

A convenience sampling of all class members who met the criteria for the study.

### Preparation for data collection

The researchers had audience with the entire class and discussed the study procedure, process, import and expected date for commencement/conclusion of the study. Clarifications on any grey areas were sought.

### Ethical consideration/approval

Ethical clearance was obtained from the Institutional Health Research Ethical Committee of the Jos University Teaching Hospital. Participants’ anonymity and confidentiality were maintained in accordance with the Helsinki Declaration.

### Data collection instrument

Weights of all the participants were measured using a brand new calibrated digital bath room weighing scale while heights were measured in metres on a standardised calibrated wall which could be substituted with a standard measuring tape in dire emergencies/the critically ill. All was without shoes, minimal clothing for weight and less of hair inclusion for height.

### Data collection/procedure

All consecutive participants were enrolled for the study at the College Lecture Hall 10/09/2015. Data was generated from their biodata, measured heights to the nearest 0.01 m which we used a standardised calibrated wall while their weights to the nearest 0.01 kg using a brand new calibrated bathroom digital weighing scale of 120 kg capacity model number BR 9011, made in China. Measurements were recorded without shoes with minimal clothing during weighing while in measuring heights we adopted measures that involved less hair inclusion. Initials were used to conceal identity.

The result was subjected to statistical analysis.

The estimated weight was obtained utilising the formula: estimated body weight (eBW) in kg = (*N*−1)100 where ‘*N*’ is the measured height in metres that can alternatively be measured using the measuring tape in dire emergencies/the critically ill. Weights within ± 10% for small and large frames of the mean percentage error (measure of accuracy) considered as the acceptable permissible estimate error (APEE) margin.

### Data analysis

The statistical analysis was performed using SPSS version 21.0, Chicago, IL, USA.

This commenced by comparing the descriptive statistics of the two measures of weight estimation.

Their body mass index(BMI) were computed both for the actual and estimated body weights and were tabulated to compare if they fell within the current classification of weights and obesity as proposed by the International Obesity Task Force (IOTF) cut-off points for BMI [16]. Positive/negative predictive values were computed to establish accuracy of the estimate. However, to establish relationship/association of the two variables, we utilised (a) the Pearson correlation coefficient (*r*) at 99% confidence interval (two-tailed) to compute for the existence of/degree of a relationship between actual body weight (ABW) and estimated body weight (eBW); a *P* ≤ 0.01 was considered significant. (b) The linear regression analysis at 95% confidence interval was utilised to establish the degree/strength of linear relationship between weight and height using the regression coefficient (*r*^2^)— the coefficient of determination, a *P* ≤ 0.05 was considered significant. (c) Furthermore, accuracy of the estimate was confirmed by finding the mean percentage error of the difference between the mean eBW and the ABW. The percentage weight accuracy of ± 10% was regarded as acceptable variation for small frame and large frame respectively [16]. (d) While the Bland-Altman plot and Cohen’s kappa statistic measure (i) bias-the difference between the estimated (eBW) and the measured (ABW). (ii) Accuracy—mean percentage error of the difference between eBW and ABW. This measures the degree of closeness between results of the calculated (eBW) and the true value (ABW). This is a qualitative concept. (iii) Precision (SD of bias) = [100(eBW − ABW ÷ ABW] describes the agreement of a set of results among themselves. This relates to the reliability, reproducibility and repeatability of results under unchanged conditions; and iv) limits/strength of agreement of the two estimates. The Bland-Altman scatterplot is a graph of the percentage of the difference between the two measures of mean weight estimation against average of the means of the two measures to compare if they lie within 95% ± 2 s [17] or bias ± 1.96 SD [18]. The results were presented in simple descriptive format, tables, figures and diagrams.

Ethical approval for the study was obtained from our Institutional Health Research Ethical Committee of the Jos University Teaching Hospital, Jos, Plateau state, Nigeria.

The study observed the Helsinki Declaration.

## Results

Analysis of 122(55.5%) adults (population size = 220) with 86 males and 36 females (M:F = 2.4:1) was performed. Their ages ranged 21 years–38 years and height = 1.55 m–1.95 m. The actual body weight (ABW) range was 48.0 kg–91.0 kg, mean = 65.3 kg ± 9.7 kg and standard error(S.E.) = 2.0 while the estimate body weight (eBW) = 55 kg–95 kg, mean = 69.1 kg ± 8.4 kg and S.E. = 1.5 (Tables 1 and 2).

**Table 1:** Height range (in metres) with their respective actual body weights’ mean, standard deviation and standard error

Height range (m)	Frequency	Mean wt (kg)	Std. deviation	Std. error
1.55–1.59	12	56.67	6.71	1.94
1.60–1.64	34	60.97	7.39	1.26
1.65–1.69	17	60.35	6.15	1.49
1.70–1.74	22	66.55	7.98	1.70
1.75–1.79	20	71.60	8.67	1.94
1.80–1.84	13	76.69	10.15	2.82
1.85–1.89	1	72.00	0.00	0.00
1.90–1.95	3	69.33	8.33	4.80
Total	122	535.16	55.37	15.96

Analysing Table 1; it can be observed that the mean weights of both the ABW and eBW in all the height range classes and their total values are very close. Furthermore, the standard deviation and standard error for the two weight estimates correlate closely and of small value signifying a better estimate

**Table 2:** Height range (in metres) with their respective estimated body weights’ means, standard deviation and standard error

Height range (m)	Frequency	Mean wt (kg)	Std. deviation	Std. error
1.55–1.59	12	56.75	6.83	2.34
1.60–1.64	34	62.21	7.45	1.43
1.65–1.69	17	66.47	6.42	1.50
1.70–1.74	22	71.77	8.26	1.41
1.75–1.79	20	75.80	8.97	1.36
1.80–1.84	13	80.69	10.36	1.32
1.85–1.89	1	85.00	0.00	0.00
1.90–1.95	3	92.33	9.13	2.52
Total	122	591.02	57.42	11.88

Analysing Table 2; it can be observed that the mean weights of both the ABW and eBW in all the height range classes and their total values are very close. Furthermore, the standard deviation and standard error for the two weight estimates correlate closely and of small value signifying a better estimate

**Fig. 1:**
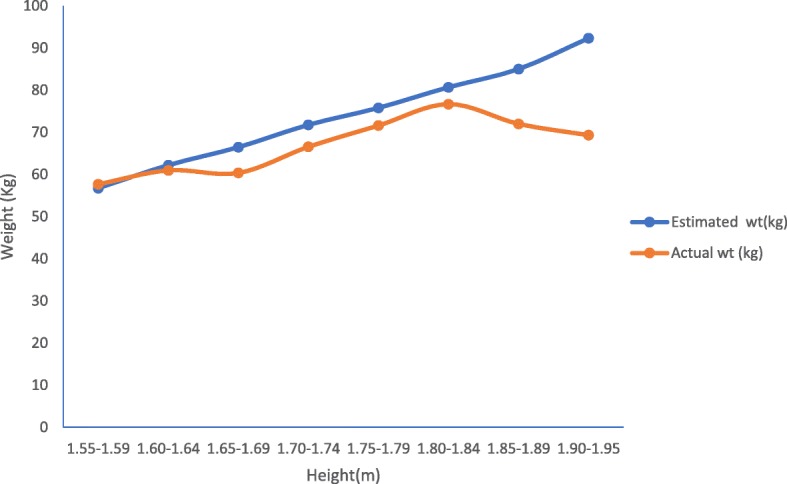
Linear regression graphs for both ABW and eBW

**Fig. 2:**
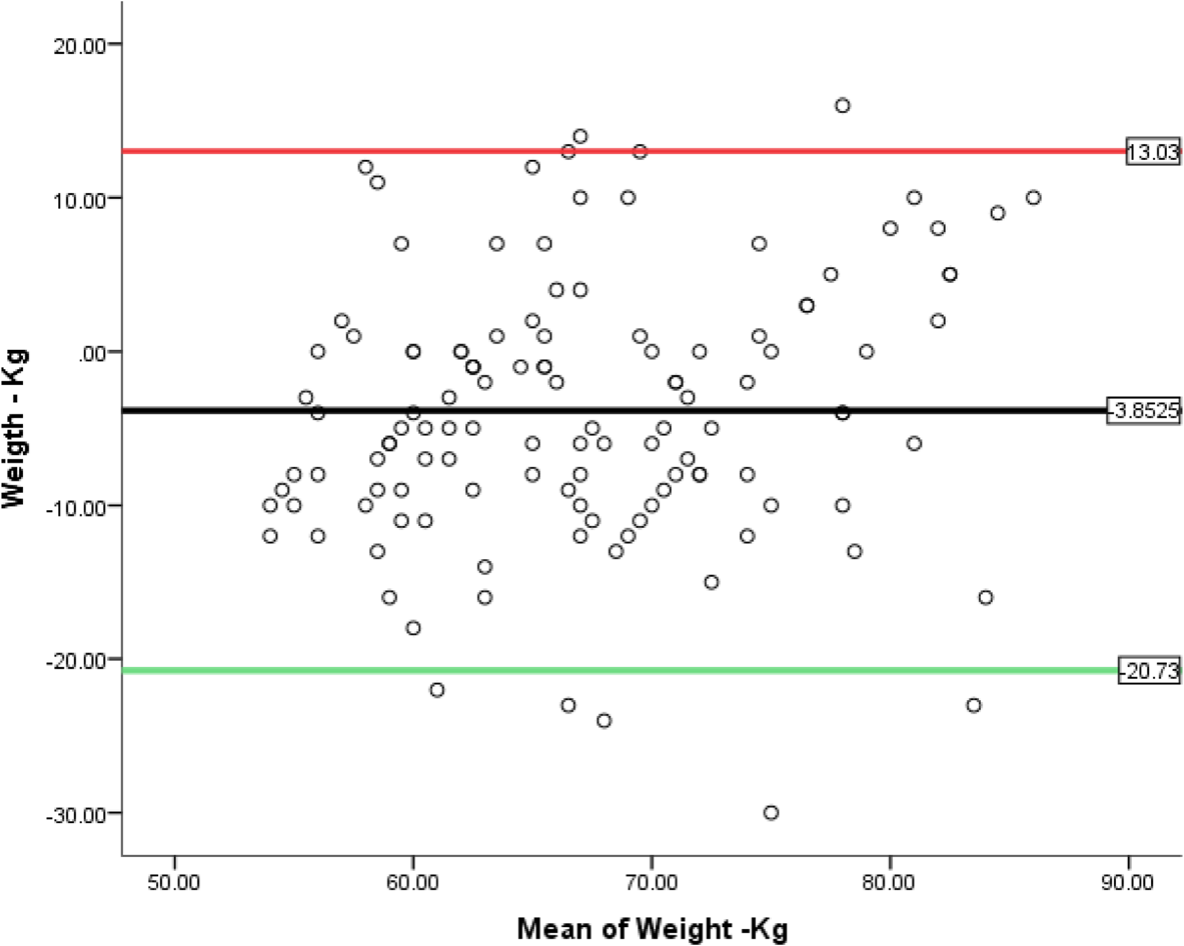
The Bland-Altman plot

The actual BMI (ABMI) range = 16.6 kg/m^2^–29.8 kg/m^2^ while that of the estimate BMI (eBMI) = 23.0 kg/m^2^–25.0 kg/m^2^. Based on BMI classification, ABW; 6 (4.9%) were under weight, 94 (77.1%) had normal weight while 22 (18.0%) were overweight. However, for eBW, none was underweight, 99 (81.1%) had normal weight while 23 (18.9%) were overweight with a positive predictive value of 94.7% for the ‘normal’ category and 95.5% for ‘overweight’. None had any of the three obesity classes (Table 3**)** implying a negative predictive value of 100%. For association/relationship between the two variables, (a) the Pearson correlation statistics at 99% confidence interval yielded a Pearson’s correlation coefficient (*r*) = + 1 (*P* = 0.000) which is strongly significant (Tables 4 and 5). (b) Linear regression analysis which established a perfect dependence of weight on height as displayed in the linear regression graph (Fig. 1) with a regression equation of *y* **=** 4.877x + 51.92 and a coefficient of determination (*r*^2^) = + 1 at 95% confidence interval (*P* = 0.000), which is strongly significant. Table 6. (c) Furthermore, Table 7 is the coded mean percentage error of the differences between eBW and ABW—a measure of accuracy of the two estimates shows 86.1% of the estimated weights fell within the acceptable permissible error (APEE) margin, which is ± 10% implying a ‘good agreement’. (d) While the bias/accuracy, precision and the degree of agreement of the two estimates was established by Bland-Altman’s (B&A) plot (Fig. 2) and the Cohen’s kappa statistic. Analytical cross-tabulation of ABMI and eBMI was also made (Tables 8 and 9). The B&A plot shows a good agreement as a considerable proportion of the estimates concentrated within the ± 10% considered as the APEE margin which was within the acceptable 95% CI ± 2 s (1.96 ± 2 s = − 16.1239 to + 20.0439). [See Table 4 where the average of the standard deviation of the two estimates (s) = 9.04195, while value for 95% CI = 1.96.] *P* = 0.000, while (e) the Cohen’s kappa statistical analysis produced a kappa value of 0.618, meaning a good agreement.

**Table 3:** Body mass index (BMI) classification of weights comparing the actual with the estimated weights

BMI (kg/m^2^)	Classification	Actual weight (kg)		Estimated weight	
		Frequency	Percentage	Frequency	Percentage
< 18.5	Underweight	6	4.9	–	–
18.5–24.5	Normal	94	77.1	99	81.1
25.0–29.5	Overweight	22	18.0	23	18.9
30.0–34.5	Obesity class 1	–	–	–	–
35.0–39.5	Obesity class 2	–	–	–	–
≥ 40	Obesity class 3	–	–	–	–
Total		122	100.0	122	100.0

Above shows a positive predictive value of 94.7% in eBW for normal weight and 95.5% positive predictive value for overweight while a negative predictive value of 100% for the obesity classes

**Table 4:** Descriptive statistics of the two estimates

Descriptive statistics			
	Mean	Std. deviation	*N*
Actual wt	65.2623	9.68752	122
Estimated wt	69.1148	8.39638	122

The descriptive statistics’ table shown above displays the mean and standard deviation for the two estimate measures ABW and eBW which are very close

**Table 5:** The Pearson correlation statistics at 99% confidence interval (two-tailed) between actual and estimated weights

Correlations			
		Actual wt	Estimated wt
Actual wt	Pearson correlation	1	.552**
	Sig. (two-tailed)		.000
	*N*	122	122
Estimated wt	Pearson correlation	1	.552**
	Sig. (two-tailed)		. .000
	*N*	122	122

NB: Table above shows correlation coefficient of + 1 (*P* = 0.000) in both the eBW and ABW meaning the two measures of weight measurement correlate (*P* ≤ 0.01)

**Correlation is significant at *P* ≤ 0.01 level (two-tailed)

**Table 6:** Regression statistics of the estimate

Variables entered/removed^b^						
Model	Variables entered		Variables removed		Method	
1	Height^a^				Enter	
Model summary						
Model	*r*	*r* ^2^	Adjusted *r*^2^		Std. error of the estimate	
1	1.000^c^	1.000	1.000		.09078	
Coefficients^d^						
Model		Unstandardized coefficients		Standardised coefficients	*t*	Sig.
		B	Std. error	Beta		
1	(Constant)	− 100.089	.167		− 601.035	.000
	Height	100.057	.098	1.000	1017.310	.000

NB: In model summary above, *r*^2^ = + 1 at 95% confidence interval with *P* = 0.000 implies a positive perfect dependence of weight on height that is linear which is strongly significant

^a^All requested variables entered

^b^Dependent variable: estimated wt

^c^Predictors: (constant), height

^d^Dependent variable: estimated wt

**Table 7:** Coded results of the mean percentage error of the difference between eBW and ABW

Frequencies statistics					
Difference					
*N*	Valid	122			
	Missing	0			
		Frequency	Percent	Valid percentage	Cumulative percentage
Valid	− 2.00	1	.8	.8	.8
	− 1.00	4	3.3	3.3	4.1
	.00	105	86.1	86.1	90.2
	1.00	12	9.8	9.8	100.0
	Total	122	100.0	100.0

**Table 8:** Analytical cross-tabulation of the BMI classification

Cross-tabulation						
Case processing summary						
	Cases					
	Valid		Missing		Total	
	*N*	Percent	*N*	Percent	*N*	Percent
Actual wt (kg)* estimated wt	122	100.0%	0	0.0%	122	100.0%
Actual wt (kg) * estimated wt cross-tabulation (for weighted kappa statistics)						
Count						
		Estimated wt			Total	
		Underweight	Normal	Overweight		
Actual wt (kg)	Underweight	2	1	1	4	
	Normal	0	89	2	91	
	Overweight	0	12	15	27	
Total		2	102	18	122

**Table 9:** Cohen’s kappa statistics

Symmetric measures				
Weighted kappa statistics	Value	Asymptotic standardised error^a^	Approximate T^b^	Approx. sig
Measure of agreement kappa	.618	.084	7.824	.000
*N* of valid cases	122			

NB: Table above demonstrates a Cohen’s kappa coefficient value of 0.618 which implies a ‘good agreement’

^a^Not assuming the null hypothesis

^b^Using the asymptotic standard error assuming the null hypothesis

## Discussion

The cohort population sample size which constituted 55.5% of the study population is in agreement with that in a study on assessing the accuracy of common paediatric age-based weight estimation formulae and the weight approximation in stroke before thrombolysis study, the so-called WAIST study [18, 19] which was an 11-month prospective observational dose-finding study in Germany undertaken for weight estimation in stroke patients receiving the thrombolytic, Alteplase; this was done on a sample size of 109. Furthermore, the demographic characteristics of the study cohort which included both gender with a male to female ratio of 2.4:1 could be said to have taken into account the gender factor in weight estimation while the age range of 21 years–38 years confirms the data being from adults which excludes pubertal participants because of transitional dynamics during puberty. In corollary, a height range of 1.55 m–1.95 m fell within the height for the study. Heights of 1.2 m–2.1 m have been described as the average heights for a population that follows a normal distribution [20–22] and therefore results obtained from such could be treated as being valid.

Analysing the descriptive statistics; the mean, the standard deviation and the standard errors for the various height categories for both ABW and eBW mirror each other. Important to note is the small-sized standard error of 2.0 for ABW and 1.5 for eBW. Standard error is the measure of uncertainty in a sample statistic. Reliability of the estimates can also be expressed in terms of the standard error of measurement. It is an estimate of how often you can expect errors of a given size.

The accuracy of the technique was established by the mean percentage error in which 86.1% were within ± 10% and the BMI classification for both the ABW and the eBW which produced positive predictive values of 94.7% and 95.5% for normal and overweights, respectively, and a negative predictive value of 100% for all categories of obesity which is a better result compared with a study [20]. Positive and negative predictive values (PPV and NPV respectively) are the proportions of positive and negative results in statistics and diagnostic tests that are true positive and true negative results, respectively. The PPV and NPV describe the performance of a diagnostic test or other statistical measure. A high result can be interpreted as indicating the accuracy of such a statistic [23].

Furthermore, the correlation statistic at 99% confidence interval of the ABW and eBW yielded a correlation coefficient(r) of + 1 (*P* = 0.000) which describes a perfect positive correlation between the two measures of weight estimation which is strongly significant and which in addition confirms a dependence of weight on height demonstrable by a positive linear regression graph with a coefficient of determination (*r*^2^) of + 1(*P* = 0.000). The coefficient of determination indicates the proportion of the variance in the dependent variable that is predictable from the independent variable being strongly significant (*P* = 0.000).

Correlation coefficient is a statistical summary of the relation between two variables. The main result of a correlation called the correlation coefficient (*r*) is computed as the ratio of covariance between the variables to the product of their standard deviations**.** The numerical value of ‘*r’* ranges from − 1.0 to + 1.0. This enables us to get an idea of the strength of linear relationship between the variables. The closer the coefficients are to + 1.0 or − 1.0, the greater the strength of the linear relationship is. When two social, physical and biological phenomena increase or decrease proportionately and simultaneously because of identical external factors, the phenomena are related positively; however, if under same condition while one increases and the other decreases, the phenomena are negatively correlated. Usually, a linear regression study is performed together with correlation measurement. Actually, linear regression can be calculated only if a correlation exists and correlation coefficient can be interpreted only if the *P* value is significant. Linear regression finds the best line that predicts one variable from the other. Linear regression quantifies goodness of fit with *r*^*2*^, the coefficient of determination. Correlation describes linear relationship between two sets of data but not their agreement. Similarly, *r*^*2*^ only tells us the proportion of variance that the two variables have in common. This result is better than that in a similar study [24, 25]. Though this formula tends to produce estimates slightly higher than the actual body weight, but overall, a significant proportion was within the ± 10% mean percentage error referred to as the acceptable permissible estimate error margin. Marginal over-estimation is also a finding with the Luscombe and Owen’s formula in general use today in paediatrics globally which are currently in use in Advanced Paediatric Life Support Protocol [13, 14].

In 1983, Bland and Altman (B&A) proposed a further analysis, based on the quantification of the agreement between two quantitative measurements by studying the mean difference and constructing limits of agreement [26]. We therefore generated the B&A plot to establish the degree of agreement of the two estimates within the established limits of 95% CI ± 2 s considered acceptable, which was strongly significant(*P* = 0.000). The overall picture infers that over 75% of the novel technique’s estimates concentrated within ± 10% mean percentage error considered as acceptable permissible estimate error and lies within the 95% CI ± 2 s which is − 16.1239 to + 20.0439. These imply a good agreement between the two measures of weight estimation; ABW and eBW. This is a far better estimate recorded compared to a study with 69% of the estimate within ± 10% [27]. Additionally, degree of agreement of the two estimates was further confirmed by the Cohen’s kappa statistic that yielded a kappa coefficient of 0.618 implying a good agreement. The kappa statistic also confers reliability of the estimates. This is better than a Cohen’s kappa coefficient of 0.54 reported in a study on the use of the RAMA Ped card in weight assessment in the paediatric age group [28]. Kappa values less than 0.20 are considered as ‘poor’, between 0.21 and 0.40 as ‘fair’ agreement between 0.41 and 0.60 as ‘moderate’ agreement, between 0.61 and 0.80 as ‘good’ agreement, and between 0.81 and 1.00 as ‘excellent’ agreement [29, 30].

## Limitations

(i) The estimated body weights are not in decimals as the tool used for height measurement can only give estimates to not more than two decimal places. (ii) The study yields the ideal weight estimate therefore cannot record underweight. (iii) The formula may not be suitable for adults < 1.20 m or > 2.00 m of height. (iv) The sample size may be a drawback; thus, the need for further large population studies in the future. (v) The performance of the equation would be dependent on the prevailing rates of obesity in a population. However, we are aware, in pharmaceutical drug formulations, the per kilogramme body weight drug dosaging have a range with a minimum and maximum dose. In this circumstance, user discretion is advised in which the maximum dosage in the obese be in relation to the estimated (ideal) weight for the corresponding height of the index case; thus, making the formula still valuable in obese patients. Severe weight loss associated with chronic illnesses is beyond the scope of this study which may be an area for future studies. Our formula: eBW = (*N* − 1)100 was derived through a trial-and-error with the power of observation, reinforced by available data that was validated statistically; a probable mathematical formula fine-tuning to cover for some of the limitations may be required in the future. This is an area for future research as well.

## Conclusion

This unprecedented statistical characterisation of the two weight estimate measures to have a good agreement scientifically proposes the utility of our method with the formula eBW(kg) = 100(*N* − 1) in critical care and ATLS protocol.

## Abbreviations

‘*r*’: Pearson’s coefficient of correlation
‘*r*^2^’: Coefficient of determination/regression coefficient
ABMI: Actual BMI
ABW: Actual body weight
APEE: Acceptable permissible estimate error
ATLS: Advanced trauma life support
B&A plot: Bland-Altman’s plot
BMI: Body mass index
CI: Confidence interval
eBMI: Estimated BMI
eBW: Estimated body weight
IOTF: International Obesity Task Force
NPV: Negative predictive value
NSRS: Nigerian Surgical Research Societies
PAFOS: Pan African Federation of Oto-laryngological Societies
PPV: Positive predictive value
RAMA Ped Card: RAMATHIBODI Paediatric emergency drug card
S.E.: Standard error
SD or S: Standard deviation
SPSS: Statistical Package for Social Sciences
WAIST study: Weight Approximation In Stroke Before Thrombolysis study
